# ALS and Oxidative Stress: The Neurovascular Scenario

**DOI:** 10.1155/2013/635831

**Published:** 2013-12-03

**Authors:** Akshay Anand, Keshav Thakur, Pawan Kumar Gupta

**Affiliations:** Neuroscience Research Lab, Department of Neurology, Post Graduate Institute of Medical Education and Research, Sector 12, Chandigarh 160012, India

## Abstract

Oxidative stress and angiogenic factors have been placed as the prime focus of scientific investigations after an establishment of link between vascular endothelial growth factor promoter (*VEGF*), hypoxia, and amyotrophic lateral sclerosis (ALS) pathogenesis. Deletion of the hypoxia-response element in the vascular endothelial growth factor promoter and mutant superoxide dismutase 1 (*SOD*1) which are characterised by atrophy and muscle weakness resulted in phenotype resembling human ALS in mice. This results in lower motor neurodegeneration thus establishing an important link between motor neuron degeneration, vasculature, and angiogenic molecules. In this review, we have presented human, animal, and *in vitro* studies which suggest that molecules like *VEGF* have a therapeutic, diagnostic, and prognostic potential in ALS. Involvement of vascular growth factors and hypoxia response elements also highlights the converging role of oxidative stress and neurovascular network for understanding and treatment of various neurodegenerative disorders like ALS.

## 1. Introduction

**Figure 1:**
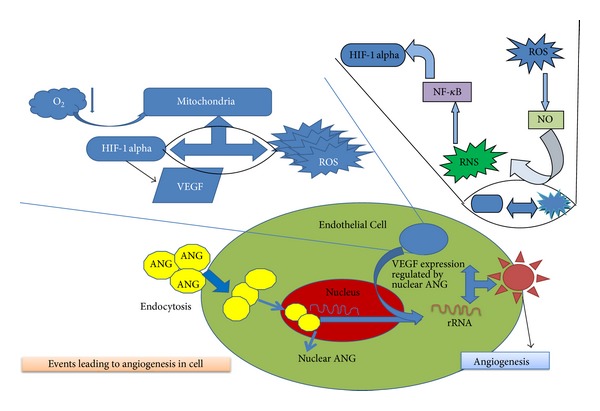
The role of hypoxia in stimulating the *VEGF* through an activation of HIF-1 alpha element. HIF-1 alpha gets activated in deficiency of oxygen in mitochondria leading to creation of oxidative stress. This involves the formation of reactive oxygen species which on reaction with free nitrogen forms NO ultimately leading to reactive nitrogen species (RNS). This RNS further activates NF-*κ*B pathway which ultimately leads to activation of HIF-1 alpha factor. The activated form of HIF-1 alpha further leads to *VEGF* activation thus leading to angiogenesis.

At the developmental stages, the establishment of a neurovascular network, outside CNS, is crucial to the subsequent brain and spinal cord development. Molecules deserving special attention in the course of development and maintenance of neurovasculature include *VEGF* (especially *VEGF*-A)/*VEGF* receptors, Notch, ephrin, semaphorins/plexin receptors, latent transforming growth factor *β*'s [*TGF*β*'s*], and TGF*β* receptors, *α*v*β*8 integrin, *neuropilins*, and FGF1 [1–3]. Any dysregulation in the pathways having the above mentioned factors (responsible for angiogenesis) which contributes to the development of this communication network has serious consequences manifesting in the form of CNS disorders. Hence angiogenesis is required for vasculature development and is governed by the gene expression of vascular molecules [4]. Abnormal expression and reduced levels of *VEGF* have been explored to account for devastating disorders of the CNS, especially in studies focused on ALS, which is designated by motor neuron degeneration and is fatal in nature [5]. Genetic studies in a transgenic mouse and rat model of ALS with mutated superoxide dismutase 1 SOD1^G93A^ have indicated that inhibition of hypoxia response element (HRE) in the *VEGF* gene promoter may promote motor neuron degeneration (since HRE is responsible for inducing angiogenesis through *VEGF* as shown in Figure 1) whereas administration of *VEGF* prolongs survival [6]. Hence, here we review the role of neurotrophic and angiogenic factors like *VEGF* in the pathogenesis of ALS. 

## 2. ALS: A Fatal Disease of the Motor Neurons

Motor neuron disease (MND) defines conglomerate of related and progressive degenerative disorders characterized by selective degeneration of upper motor and lower motor neuron located in the motor cortex and brain stem and spinal cord, respectively [4]. The disease may either affect lower motor neuron (progressive muscular atrophy) or upper motor neurons (primary lateral sclerosis) or both upper/lower motor neurons (amyotrophic lateral sclerosis); however, careful pathological and clinical studies in MND have shown that extra-motor parts of the central nervous system are also affected. ALS is the most severe MND where selective degeneration of motor neurons leads to atrophy of voluntary muscles followed by paralysis and may prove fatal [5]. Mechanisms of selective degeneration of motor neurons in ALS are obscure. Largely, ALS symptoms include weakness of muscles, especially those in the hands, arms, and legs with or without dysarthria and dysphagia. Fasciculation or muscle twitching is also an important clinical finding [7]. 

## 3. ALS: Contributing Factors

ALS occurs in both sporadic and familial form at an incidence varying between 0.4 and 2.6 for every 100,000 individuals and a prevalence rate of 4–6 per 100,000 population per year [8]. The etiology of ALS has been elusive and believed to be multifactorial. Though causes of most cases of ALS are unknown, major factors include genetic factors like point mutations in superoxide dismutase 1 (SOD1) gene accounting for around 20% of familial ALS (fALS) cases [9]. The purely lower motor neuron (LMN) degeneration variant of ALS shows missense mutations in *CHMP2B* (charged multivesicular protein 2B; involved in cellular transport). In 10% cases of ALS, patients with *CHMP2B* mutations are shown to have lower motor neuron degeneration. Apart from this, other genes like vesicle-associated membrane protein B (*VAPB*) (which is involved in providing unfolded protein response to endoplasmic reticulum), senataxin (*SETX*) (gene present in central nervous system involving brain and spinal cord as well as muscle and play major role in DNA repair to maintain integrity of cell), and *dynactin 1* (involved in cellular transport during cell division and specially in axonal transport of nerve cells) with mutations have been shown to play role in aggregate formation and hampering the normal activity of the motor neurons thus contributing to the pathogenesis of ALS overall in subject's body [10–14]. Genes encoding angiogenin (*ANG*) having missense mutations have also been involved in the pathogenesis of ALS. Angiogenin, like *VEGF*, is produced in response to hypoxia and plays a role in neovascularisation as shown in Figure 1. Its importance further stems from the fact that it can regulate the expression of *VEGF* [15, 16]. Hypoxia takes place when oxygen availability is low in cell due to which the mitochondria produces ROS species which in turn reacts with nitric oxide (NO) to produce reactive nitrogen species RNS and activates HIF-*α* pathway through NF-*κ*B pathway resulting in stimulation of *VEGF*. The expression of this *VEGF* is dependent on the nucleolar *ANG* which directly helps in stimulating the proliferation of epithelial cells and helps in angiogenesis [17]. However, this hypothesis raises a question whether angiogenin crosses the blood brain barrier or is retained in cerebrospinal fluid [18].

Apart from genetic factors, the presence of insoluble intracellular protein aggregates in motor neurons and reactive astrocytes are considered as the hallmarks for the disease (Figure 3). [19]. The other factors include glutamate toxicity [20], lack of trophic growth factors [6, 21], autoimmunity [22], toxin [23], and susceptibility of motor neurons to neuro-degeneration because of their large size and high energy demands [24]. 

Currently, there is no treatment that could substantially alleviate the disease burden because of incomplete understanding of ALS etiology. Food and Drug Administration (FDA) has approved only single drug for the treatment of ALS, a glutamate antagonist that is Riluzole [25, 26]. Riluzole has also been studied as a potential inhibitor of *VEGF* induced endothelial cell proliferation under both *in vitro* and *in vivo* conditions [27]. Its neuroprotective effect via sodium channel blockage is brought about by the fact that this mechanism increases resistance to hypoxia through a reduction in energy demands (a decreased cerebral glucose consumption) [28].

## 4. **VEGF**: The Neurotrophic and Angiogenic Family 

*VEGF*A gene in humans is positioned at chromosome 6p21.3 with eight exons and is expressed as several isoforms of different amino acid chain lengths because of alternative splicing (*VEGF*_121_, *VEGF*_145_, *VEGF*_165_, *VEGF*_183_, *VEGF*_189_, *VEGF*_206_) [29] that differ in their ability to bind heparin, *neuropilin*-1 (NP-1), and *neuropilin*-2 (NP-2). Two classes of receptors for *VEGF* are the tyrosine kinase and the nontyrosine kinase receptors. *VEGF*R1 (Flt-1 (fms-related tyrosine kinase 1)), *VEGF*R2 (KDR/Flk-1 (kinase insert domain receptor/fetal liver kinase-1), and *VEGF*R-3 (Flt-4) are three structurally related receptors present in tyrosine kinase class V, whereas *neuropilin*-1 (NP-1) and *neuropilin*-2 (NP-2) are part of nontyrosine kinase receptors. *VEGF* binds to NP 1 and 2 and *VEGF*R1 and 2 but not to *VEGF*R-3 as the latter one is not a receptor for *VEGF*. Studies indicate that for transmission of critical angiogenic signals in response to *VEGF VEGF*R2 plays the role of key mediator [30]. However in case of *VEGF*R1 the major function is prevention of *VEGF* binding to *VEGF*R1 thought to be done by a virtue of “decoy receptor” to negatively regulate angiogenesis [31]. *Neuropilins* (NP1 and 2) whose primary location is in central nervous system are described as receptor for collapsin/semaphorin family, which are responsible for controlling neuronal cell guidance [32, 33]. For *VEGF*165 and a coreceptor of *VEGF*R2 *Neuropilin-1* (NP-1), it is a specific receptor whereas *Neuropilin*-2 (NP-2) binds *VEGF*165 and *VEGF*145 in isoform specific manner. *VEGF* is the part of genes which accommodate placental growth factor (PLGF), *VEGF*B, *VEGF*C, *VEGF*D, and *VEGF*E including *VEGF*-A, out of which lymphatic vessels development is affected by *VEGF*-C [34]. Recent evidence from studies also indicates that neural cells are directly affected by *VEGF*-A, *VEGF*-B and *VEGF*-C [35]. In ALS, *VEGF* has been studied as an important member of gene families impacting the pathology of disease. 

## 5. **VEGF**: Molecular Risk Factor in ALS

The lack of trophic (growth) factors has been hypothesized as probable cause of ALS. Since growth factors are neurotrophic and help in growth, survival, and maintenance of neuronal cells. The hypoxia response brings together a cascade of events involving angiogenic and inflammatory factors (Figures 1 and 3). Studies have focussed on predicting/correlating disease state with changing levels of such factors in body fluids even though these have been conducted utilising heterogeneous controls.

*VEGF* and its receptors are reported to be localised in neurons and astrocytes [36, 37] which, in case of ischemia or spinal cord injuries, provides neuroprotection and stimulates neuronal growth. Decreased *VEGF* levels may impair perfusion and induce ischemia of motor neurons, other than depriving cells of important survival and neuroprotective signals which are *VEGF* dependent [6].

Cronin et al. reported elevated levels of serum angiogenin, but no change in serum *VEGF* levels was observed. The authors also failed to observe any correlation between serum angiogenin and *VEGF* levels [16]. In another study, the patients with limb onset and long duration of ALS showed higher concentration of CSF *VEGF* as compared to those with bulbar onset of ALS and patients with short duration illness, respectively [38]. It may be possible that significant increase in cerebrospinal fluid (CSF) *VEGF* levels may have protective role against over-excitation of motor neurons (excitotoxicity). This overexitation may be mediated by excessive accumulation of glutamate at synaptic cleft in patients with limb onset of ALS and those with long duration of the disease, since it was suggested that the increased levels of *VEGF* account for a compensatory mechanism and may be required to stabilize neuronal excitation [39]. The rationale was further supported by Bogaert et al. who reported that *VEGF* protects motor neuron against excitotoxicity by upregulating Glutamate receptor 2 [40]. Significantly, lower baseline CSF *VEGF* levels in case of patients with ALS in comparison to normal controls and neurologic controls during early phase of disease have been observed, suggesting the possible link of ALS pathogenesis with *VEGF* gene regulation [41]. 

Moreau et al. demonstrated that hypoxaemic ALS patients had lower *VEGF* levels in CSF from normoxaemic ALS patients. This happened due to an early defect in hypoxia induced factor-1 (HIF-1) mediated regulation of *VEGF*. In contrast, higher levels of *VEGF* in CSF were demonstrated in hypoxaemic neurological controls than normoxaemic neurological controls. Hypoxaemia severity in ALS is explained by dysregulation of *VEGF* in ALS. This association of *VEGF* expression and hypoxia (Figure 1) in ALS introduced a concept of incongruous response [42]. Nagata et al. failed to reproduce the above results as no significant difference was observed in CSF *VEGF* levels between ALS patients, normal controls, and controls with other neurological disorders [43]. It was argued by Cronin and coworkers that the conflicting reports of elevated, normal, and decreased *VEGF* might have resulted from different study designs and ELISA kit employed with varying diagnostic criteria of ALS patients, diverse clinical details of ALS patients including definite and probable forms of disease [16]. In a unique histochemical study, a markedly elevated level of *VEGF* was detected in the skin of ALS patients when compared with normal subjects suggesting a positive correlation of *VEGF* levels in skin and severity of ALS patients [44]. The finding suggests systemic dysregulation of *VEGF* expression in ALS. Recently, it has been observed that elevated levels of *VEGF*A in CSF, serum, and peripheral blood mononuclear cells may account for substantially prolonged life span of Indian ALS patients as compared to their Western counterparts [45–47]. Surprisingly, longer survival is shown in Indian ALS patients after onset (~9 year) of ALS [45, 46, 48, 49]. Further, reduced levels of soluble *VEGF*R1 (s*VEGF*R1), an inhibitory receptor of *VEGF*, have been observed in these patients, supporting the neurotropic nature of *VEGF* [50]. However, these results need confirmation in comparable Caucasian ALS population. 

## 6. ALS: **VEGF** and Oxidative Stress

**Figure 2:**
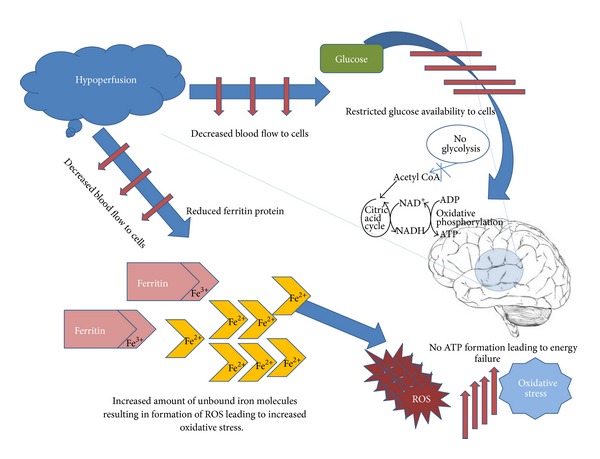
Role of hypoperfusion in elevation of oxidative stress and energy failure. As hypoperfusion reduces blood flow towards cells resulting in reduced ferritin Fe^3+^ protein, it releases unbound iron Fe^2+^ molecules resulting in formation of ROS thus increasing the oxidative stress. Hypoperfusion also leads to unavailability of glucose to brain cells thus leading to energy failure.

Lowering of *VEGF* levels places neural tissue at the risk of limited perfusion thus making way for motor neuron degeneration [51]. This degeneration is a direct consequence of the fact that the deficient oxygen and glucose levels created as a result of decreased vascular perfusion can hardly meet the energy demands of motor neurons [52]. Oxidative stress due to hypoperfusion has been reported in cases of other neurodegenerative disorders such as Alzheimer's disease [53]. Oxidative stress is one of the outcomes of hypoperfusion apart from energy failure as blood is known to carry several vital components essential for cell survival including glucose and ferritin. As glucose is able to readily cross blood brain barrier (BBB), the deficiency of blood flow leads to reduced supply of glucose to brain resulting in limited energy production for cells. Similarly, the deficiency of ferritin, which is responsible for binding of free iron, results in formation of reactive oxygen species as shown in Figure 2 [54]. At least one study has reported that the variable levels of *VEGF* lead to altered ferritin levels [55]. Therefore, it is safe to say that oxidative stress deserves special significance in the pathogenesis of neurodegenerative diseases like ALS since motor neurons are particularly susceptible to oxidative damage. 

This significance is born out of the fact that the first evidence of association between ALS pathology and *VEGF* came when Oosthuyse et al. created homozygous *VEGF* (*VEGF* ^*δ*/*δ*^) knock-in mice by introducing homozygous mutation of hypoxia response element (HRE) in the *VEGF* gene promoter to study angiogenic property of *VEGF*. They observed that almost 60% of mice did not survive before or around birth due to vasculature aberrations in lungs. The 40% who survived began to develop symptoms like classical ALS around five months of age [6]. This unusual finding compelled researchers to explore significance of growth factors in pathology of ALS utilising a variety of tools such as those discussed below.

### 6.1. Autopsy Based Studies

Spinal cord tissue analysis of ALS patients has revealed elevated dendritic cell marker transcripts (like CD83) and monocytic/macrophage/microglial transcripts [56], expression of cyclooxygenase-2 (*COX-2*) [57], connective tissue growth factor (CTGF) [58], monocyte chemoattractant protein-1 (*MCP1*) [56] and *VEGF* receptor (*VEGF*R)-1 [59], and activity of glutamate dehydrogenase (*GDH*) accompanied by reduced levels of glutamate and aspartate [60].

**Figure 3:**
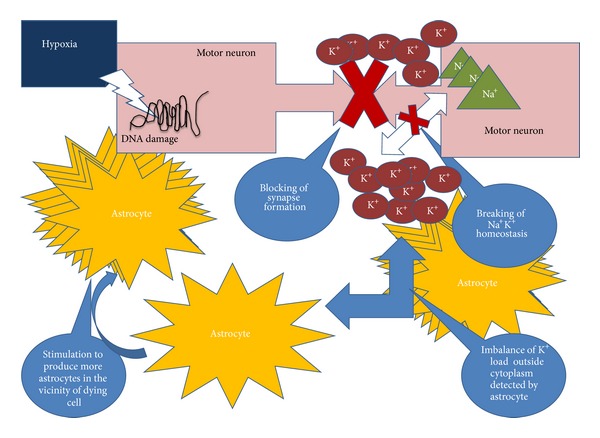
Hypoxia induced mobilisation of astrocytes. Astrogliosis is the result of aggressive increase of astrocytes number in the vicinity of damaged neuron cell. Synapse formation is hampered when there is neuronal damage thus leading to breakdown of Na^+^K^+^ homeostasis. This K^+^ concentration is detected by the astrocytes.

The increase in CTGF expression is explained by the fact that CTGF plays an important role in astrogliosis which is often seen as a consequence of hypoxic conditions and is therefore a pathological hallmark of ALS [58]. As depicted in Figure 3 astrogliosis is the result of aggressive increase of astrocytes number in the vicinity of damaged neuron cell. Hypoxia generally induces damage in the DNA of the neuronal cells. Since the neuronal damage has taken place its normal activity of synapse formation is hampered affecting the Na^+^K^+^ activity in those cells leading to breakdown of Na^+^K^+^ homeostasis. This change in balance of K^+^ concentration is detected by the astrocytes. This alteration results in the activation of astrocytes by initiation of clustering around the damaged cells in order to restore the functioning of those damaged cells [61–63]. 

Gliosis is also related to the enhanced GDH activity as reported by Malessa et al. [60]. The function of the GDH is to enhance the availability of the glutamate. This glutamate further acts as neurotransmitter or gliotransmitters since it increases the availability of Ca^+^ required by glial cells to perform their normal function of providing protection, nutrition, and avoiding accumulation of any chemicals involved in synapse formation which may later lead to toxication of neuron cell. Recruitment of glial cells to the site of damage may be considered as the body's primary response to save the dying neurons [64, 65], and thus the fact may be related to the point of association of enhanced GDH activity to gliosis. The authors also suggested a disturbance in cholinergic transmission in ALS spinal cord thus contributing to the reduced amino acid levels [60]. Glutamate and aspartate amino acids are linked with the neurotransmitters in the body. They are mainly the excitatory neurotransmitters, which utilise the Na^+^K^+^ pump to maintain their flow to the postsynaptic cleft during the nerve transmission. Li and Zhuo demonstrated that cholinergic transmitters play a role in inhibiting the glutamate based transmission. Release of acetylcholine leads to the activation of the muscarinic receptors, resulting in an inhibition of AMPA receptors (also called as glutamate receptors), and it increases the nonavailability of glutamate. This evidence also supports the fact mentioned in the above study that disturbance in cholinergic transmission may lead to reduced amino acid levels [66].

*VEGF* was first measured in spinal cord and serum of ALS patients by Nygren and colleagues. Authors did not observe any significant alteration in spinal cord *VEGF* levels, but they were able to observe higher serum *VEGF* levels in ALS patients in comparison to controls similar to those later reported by Gupta et al. in case of Indian ALS patients [46]. Considering the higher levels of *VEGF* in serum suggests that the cells other than central nervous system or which are not part of CNS are involved. In case of ALS skeletal muscles are the most affected region of body. Regional ischemia, a condition in which the blood supply is halted in specific region of brain, has been reported in case of ALS [67]. Rissanen et al. observed higher levels of *VEGF* in skeletal muscles with acute phase of ischemia [68]. Thus, it was hypothesized that *VEGF* is expressed in skeletal muscles in response to hypoxia and the increase was also reflected in serum [69].

The autopsy samples depict the terminal stage of the disease and provide a reliable proof of the disease and its signatures [70].

### 6.2. Muscle Biopsy Based Studies

In contrast to the increased cyclooxygenase (*COX*) activity in spinal cord of ALS patients, as discussed above, Crugnola et al. reported *COX* deficiencies in 46% patients, based on their histochemical analysis of muscle specimens. Moreover, molecular studies and biochemical analysis on the selected specimens displaying severe *COX* deficiencies even correlated with mutations in *SOD1* and *TARDBP* genes and mitochondrial DNA defects thus pointing towards the secondary nature of *COX* deficiencies in the pathogenesis of ALS in light of the genetic nature of defects [71]. This is also confirmed by the findings of Vielhaber et al. who observed mitochondrial DNA damage in skeletal muscle, along with lowered levels of mitochondrial Mn-SOD [72]. The specific nature of mitochondrial dysfunction is further revealed by studying mitochondrial markers like citrate synthase and succinate dehydrogenase in muscle, histochemically. However, such a study by Krasnianski et al. revealed that one cannot narrow down the observed mitochondrial changes to only depict ALS but in fact view them as an indication of other neurogenic atrophies too [73]. In view of neurotrophic support provided by muscle tissue, the findings by Küst et al. depicted enhanced expression of nerve growth factor (NGF) and neurotrophins such as brain-derived neurotrophic factor (BDNF), in postmortem bicep tissue of ALS patients. Even so, externally administered neurotrophins have not shown promising results in human trials or animal models of ALS [74].

### 6.3. Polymorphism Based Studies

Increased oxidative stress implies consequent increased oxidative damage for motor neuronal DNA. Such oxidative damage of DNA is driven by the base excision repair (BER) system. One such product of oxidative damage of DNA is 8-hydroxy-2′-deoxyguanosine (8-OHdG) which is regulated by two enzymes, namely, human 8-oxoguanine DNA glycosylase 1 (*hOGG1*) and apurinic/apyrimidinic endonuclease APE1. Consequently, mutations and polymorphisms in coding area of genes coding for both of these enzymes are of interest to researchers. Concurrent oxidative stress conditions and a faulty DNA repair system are a risk factor for motor neurons.

In most studies concerning *hOGG1* Ser326Cys polymorphism levels of 8-OHdG are taken into account as 8-OHdG is the product of DNA oxidation [75]. A study conducted by Chen et al. showed the reduced activity of *hOGG1* in patients with 326 CC polymorphisms (*P* = 0.02) as compared to those with 326 SC polymorphisms (*P* = 0.05) [76]. Similar observations were made by authors in current study. In a Caucasian study, Coppedè et al. studied the distribution of allele frequencies and genotypes in sALS patients and controls for the *hOGG1* Ser326Cys polymorphism in sALS patients and controls. The authors reported a significantly increased sALS risk associated with a combined Ser326Cys + Cys326Cys genotype. However, the Ser326Cys genotype showed nonsignificant results predicting that the *hOGG1* Ser326Cys polymorphism in patient also pose a risk factor for ALS. Ser326Cys polymorphism takes place when at exon 7, position 1245 C to G substitution occurs and as a result S is substituted to C in codon 326. 

Another interesting observation (though not significant as the test group of subjects used for the study was small, more significant results can be obtained if the study with large number of patients is conducted) in the above study was the fact that sALS patients as opposed to those bearing one or two copies of the 326Cys mutant allele bearing the Ser326Ser genotype displayed lower levels of AOPP (advanced oxidation protein products; believed to be stable markers of oxidative damage to proteins) [77]. Since abnormal levels of *VEGF* are implicated as risk factor in ALS, it is evident that mice with hypoxia response element deletion in vascular endothelial growth factor gene develop features reminiscent of ALS [5] although no spontaneous mutations have been observed in HRE in ALS patients [78, 79]. Large family-based and case-control cohort of North American white subjects (*n* = 1,603) were studied for the association of sALS with promoter polymorphisms of three *VEGF* genes. *VEGF* promoter polymorphisms do not find their casual role in ALS in light of absence of their association with sALS [80]. Risk of developing ALS has been associated to *VEGF* due to alterations in sequence in the promoter region of gene. In The Netherlands, 373 patients with sporadic ALS along with 615 matched healthy controls were found to have *VEGF* promoter haplotypes. No significant association between the previously reported at-risk haplotypes and ALS was found [81]. However, in some studies ALS has been found to be associated with *VEGF* C2578A polymorphism. In a study of Chinese population by Zhang et al. 115 sALS patients with 200 healthy individuals were analyzed for C2578A polymorphism (by amplifying 2705 to 2494 bps of *VEGF* gene promoter). Reports were in disagreement to previous studies from Caucasian populations as Chinese population did not fall susceptible for ALS due to C2578A polymorphism (attributing the effect to different genetic background in Chinese population) [82]. No significant association of ALS with three common *VEGF* variations [-2578C/A, -1154G/A, and -634G/C] in original form or in haplotype combination in a recent meta-analysis study comprising of over 7000 individuals involving three North American population and eight European populations was reported. However, in males -2578AA genotype increased the risk of ALS in subgroup analyses by gender [83] in contrast to a German study which suggested that risk of ALS in case of female patients might be higher as the *VEGF* role might be gender dependent [84]. Oates and Pamphlett did not observe any alteration of functioning of motor neurons by epigenetic transcriptional silencing of *VEGF* gene by methylation [85]. Additionally, screening of regulatory sequences of *VEGF*R2 found no association of polymorphism of *VEGF*R2 gene with risk of ALS [86]. Although association of *VEGF* with ALS has been well established by culture and animal studies, evidence from genetic studies in human cohorts suggests only a minor association between *VEGF* and the risk of developing ALS.

 The role of *VEGF* involvement in ALS is questioned due to lack of association of *VEGF* genotypes and haplotypes in large meta-analysis study. Possibilities for *VEGF* role in predisposed patients to ALS cannot be ruled out. More studies are needed to discern the actual role of *VEGF* in pathogenesis of ALS. 

### 6.4. Animal Model Based Studies

#### 6.4.1. Primates

The concept of utilizing the cytotoxic properties of the extract obtained from the spinal cord of ALS patients was applied by Zil'ber et al. as early as in 1963 so as to reproduce the disease in rhesus monkeys. The authors could only conclude to a viral nature of this disease, but at the same time they recognised that the high incidence previously reported in the Chamorro tribe of Guam suggested a unique basis [87]. Another study conducted on rhesus monkeys attempted to validate the efficacy of bovine *SOD* as a therapeutic agent to compensate for the functions of the mutated form of the enzyme. SOD being a locally acting enzyme was administered intrathecally and intraventricularly so as to bypass the blood brain barrier. The injected bSOD showed commendable tolerance though its clearance was slower when compared with results obtained from rats. But the therapy when administered into a late stage FALS patient did not show promising results [88]. 

#### 6.4.2. Rodents

The neuroprotective effect of *VEGF* suggests that exogenous *VEGF* administration may prevent degeneration of motor neuron. In a SOD1^Gly93Ala^ rat model of ALS, it was shown that onset of paralysis was delayed by 17 days, improved motor performance, and extended lifespan by 22 days due to intracerebroventricular (i.c.v.) delivery of recombinant (*VEGF*). The study demonstrated the high scale effect in animal models of ALS achieved by protein delivery [89]. Intrathecal transplantation of human neural stem cells overexpressing VEGF increased the duration of survival of a transgenic ALS mouse model [90]. Similarly, mice after spinal cord ischemia showed susceptibility to paralysis in nervous tissue with reduced *VEGF*-A expression levels whereas after treatment with *VEGF*-A showed protective effect against ischemic motor neuron death [91]. These results unveil a therapeutic potential of *VEGF* for degenerating motor neurons in case of human ALS. In similar study authors investigating the protective role of *VEGF* during ischemia has shown to reduce infarct size, improve neurological performance, and enhance the survival of newborn neurons in the dentate gyrus and subventricular zone in adult rat brain with focal cerebral ischemia. Thus, *VEGF* shows acute neuroprotective effect, and prolongs survival of new neurons in the ischemic brain [92].

Zheng et al. demonstrated for the first time in Cu/Zn *SOD1* transgenic mouse model of ALS that *VEGF* delayed diseased symptoms progression and prolonged survival, suggesting the importance of *VEGF* or related compounds in the treatment of ALS patients [93]. Rats having *VEGF* treatment showed significantly improved performance up to 6 weeks after spinal cord contusion injury compared with control animals. Furthermore, the group showed that *VEGF* treated animals had increased amount of spared tissue in the lesion centre with higher blood vessel density in parts of the wound area compared to controls, proving neurogenic and angiogenic capacity of *VEGF* [94]. Enhanced expression of *VEGF* by intramuscular administration of zinc finger transcription factor in *SOD1* rats has been shown to improve functional disability [95]. Nitric oxide is known to decrease pressure in blood vessels [96], and it is possible that low *VEGF* adversely affects vasculature via changing the amount of nitric oxide released from endothelial cells, which further impairs perfusion and causes ischemic damage of motor neurons [91]. Moreover, decreased flow of blood has been observed in patients with ALS [97]. Both mechanisms may contribute to adult-onset progressive degeneration of motor neurons, muscle weakness, paralysis, and death, a typical feature of amyotrophic lateral sclerosis. It was earlier demonstrated that exposure to low levels of lead prolongs survival of ALS transgenic mouse, possibly mediated by upregulation of *VEGF*, which in turn reduces astrocytosis [98]. In another case retrograde delivery of lentivirus into mouse model of ALS prolonged survival in animals. Authors reported that lentivirus helped in stimulation of *VEGF* levels during diseased condition in animals [99]. Although in ALS animal models *VEGF* delivery has been successful, dose of delivery of *VEGF* should be adequately optimized to prevent adverse effects on the vascular system. It is possible that levels of *VEGF* higher than a certain threshold value may increase leakiness of blood vessels and modulate permeability of blood brain barrier [100] and therefore result in intrathecal accumulation of fluid. The presence of the blood breakdown product hemosiderin in and around spinal cord motor neurons supports increased leakiness and malformed blood vessels in ALS mouse models [101]. 

It must be noted that a drawback with using *SOD1* based transgenic models is that *SOD1* gene mutations represent only 20% of cases of familial ALS, which themselves represent just 10% of the total ALS cases. Therefore, remaining 90% of ALS cases, sporadic in nature, are difficult to mimic using such animal models [70].

### 6.5. Cell Culture Based Studies

Owing to a translational gap from animal models of ALS to humans, *in vitro* investigations utilising human motor neurons and astrocytes purified from the human embryonic spinal cord anterior horns allow for greater manipulations and are therefore a critical tool in discerning mechanisms pertaining to motor neuron degeneration in ALS [102]. 

The mRNA level of *VEGF* has been an important marker to analyse the role of *VEGF* in ALS. Destabilization and downregulation of *VEGF* mRNA with concomitant loss of protein expression in glial cells expressing mutant *SOD1 in vitro* are in consensus with many reports on the role of reduced *VEGF* expression in ALS pathogenesis [103]. In contrast, it was reported that hypoxia induced proteins bind and stabilize VEGF mRNA transcript resulting in increased expression of VEGF as a compensatory protective mechanism in later stages of disease [104].

The potential role of *VEGF* in preventing cell death by SOD-1 mutation has been studied in NSC-34 motor neuron cell line from mouse. Infection by adenovirus containing mutant Gly93Ala-*SOD1* was shown to increase cell death and cellular oxidative stress. However, *VEGF* showed a dose dependent resistance to oxidative damage from hydrogen peroxide, TNF-alpha, and mutant Gly93Ala-*SOD1* in NSC-34 cells treated with *VEGF*. Both phosphoinositide-3-kinase (PI3-K) and mitogen activated protein kinase (MAPK) activities in mouse NSC-34 motor neuron-like cells were activated by *VEGF* [105]. Recently, a culture study using primary culture of *SOD1* mutated rat motor neurons has shown that decrease in *VEGF* before or during motor neuron degeneration amplifies the risk of mutated *SOD1* induced toxicity in motor neurons [106]. Thus, the *in vitro* study shows *VEGF* as an antiapoptotic molecule. Overexpression of *VEGF* in the hippocampus using recombinant adeno associated virus vector in adult rats has been reported to result in improved cognition in association with approximately 2-fold increase in neurogenesis. Moreover, environmental induction of neurogenesis is completely blocked RNA interference based inhibition of *VEGF* expression. This data supports a model whereby *VEGF* acting via kinase insert domain receptor (KDR) is a mediator of the effect of the environment on neurogenesis and cognition [107]. Meng et al. investigated *in vitro* the proliferation and differentiation of subventricular zone neural progenitors of adult mouse by virtue of direct effect of *VEGF*. Downregulation of endogenous *VEGF* receptors 1 and 2, in association with reduced neural progenitor cell proliferation and enhanced neuronal differentiation, was reported as a result of high dose (500 ng/mL) of *VEGF*, whereas endogenous *VEGF* receptors 1 and 2 were significantly upregulated without increased proliferation and differentiation at low dose (50 ng/mL) of *VEGF*. Above given experiments suggest that *VEGF* regulates neurogenesis and its high dose enhances adult neural progenitor cell differentiation into neurons showing exogenous *VEGF* to exert a biphasic effect on the expression of endogenous *VEGF* receptors [108]. It has been shown that *VEGF* induces differentiation of stem cells in endothelial cells which in turn secrete various neurotrophic factors and infers a novel mechanism of neuroprotection by *VEGF* [109]. Apart from *VEGF*, recently, *VEGF*B was shown to protect cultured primary motor neurons. Further, it was observed that mutated SOD1 ALS mouse without *VEGF*B gene developed more severe form of ALS than ALS mouse with *VEGF*B [110].

## 7. **VEGF** in Blood Brain Barrier (BBB) and Blood Spinal Cord Barrier (BSCB)

**Figure 4:**
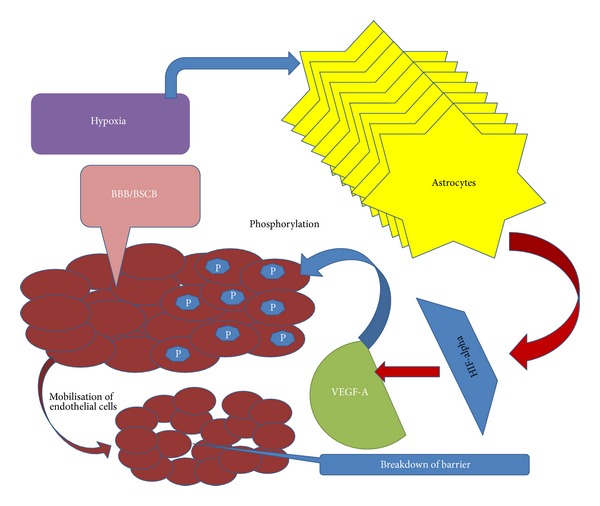
*VEGF* and permeability of blood brain barrier. Astrocytic expression of HIF-alpha and *VEGFA* leads to downregulation of claudins CLN-5 and their regulatory protein OCLN. *VEGFA*, by the virtue of tyrosine phosphorylation, downregulates the expression of CLN ultimately resulting in disruption of permeability barrier. *VEGF* induces the migration among the endothelial cells and increases the permeability to CNS.

Blood brain barrier (BBB) is the only checkpoint that stops inflammatory agents to reach central nervous system (CNS), as it contains a balanced interaction of microvascular endothelial cells and other components such as astrocytes, pericytes, neurons, and basement membrane. These components are collectively called as neovascular unit NVU. Tight junctions among NVU make the entry of undesirable components restricted to CNS [111]. BBB breakdown may lead to disruption of various biochemical reactions or may lead to accumulation of various inflammatory proteins that may aggravate the disease conditions of CNS [112–114]. Similarly, blood brain and spinal cord barrier which can be a morphological isotype for BBB performs same function in separating the spinal cord from all harmful components that may lead to diseased conditions of nervous system [115]. It has been observed that in case of human and animal model studies both the infiltration of brain and spinal cord with T cell, dendritic cells, or IgG have resulted in degeneration of motor neurons [116]. Claudins play a major role in forming tight junctions in the body among the cells to function as a barrier or act as a filter for these inflammatory factors to enter CNS [117]. Earlier studies have shown that astrocytes produce certain chemokines which play a role in attracting the dendritic cells to the CNS [118]. Recently, a link between the reactive astrocytes and disruption of these barriers has been reported. Argaw et al. tried to examine a link between astrocyte derived *VEGF*A and BBB permeability. Astrocytic expression of HIF-alpha and *VEGF*A leads to downregulation of claudins CLN-5 and their regulatory protein OCLN [119]. *VEGF*A, by the virtue of tyrosine phosphorylation, downregulates the expression of CLN ultimately resulting in disruption of permeability barrier. *VEGF* induces the migration among the endothelial cells and increasing the permeability to CNS [120] (Figure 4). However, theis link of *VEGF* is conflicting with the earlier reports in this paper regarding the protective role of *VEGF* in ALS pathogenesis.

## 8. Natural Products and Regulation of **VEGF** Expression

Naturally occurring compounds are also in current focus to examine their role in *VEGF* expression. The mechanism has been postulated to be common in all cases for those which are known to be responsible for increase in the expression of *VEGF*. All of them have been shown to affect the HIF pathway inducing the expression of *VEGF*. It is not clear how these natural compounds can be successfully translated for clinical use in near future which will need more studies. Certain extracts like *turmeric*, *gigko biloba*, and *ginseng* have been shown in mice studies to delay the disease onset or prolong survival in mice studies. However, recently a group from China reported a component *Baicalin* in the roots of plant *Scutellaria baicalensis* which enhances the expression of *VEGF* [121]. Although the HIF expression was less as compared to *VEGF*, authors reported that other transcription factors such as oestrogen-related receptors (EERs) exert their effects via *VEGF* promoters. Peroxisome proliferator-activated receptor-*γ* coactivator-1*α* (*PGC-1*α**), an important molecule independent of activator, is shown to interact with* ERR*α** [122, 123]. These *PGC-1*α** are shown to enhance expression of *VEGF* in cultured muscle cells *in vivo* in HIF independent pathway [121, 124]. In contrast, grape seed extract (GSE) is known for its antitumor properties and is shown useful in case of breast, lung, skin, or gastrointestinal cancer [125–127]. Lu and group recently showed that GSE reduced the *VEGF* expression by inhibiting the HIF expression in human breast tissue cancer cells. Authors argued that it involved the blockade of HIF expression by inhibiting AKT-3 pathway normally known for supporting the cell survival [128]. Apart from these other natural components have been shown to provide nonsatisfactory results in certain trials conducted in different human population. Vitamin E the most commonly studied antioxidant has been implicated with role of slowing down the disease progression in its severe form. Desnuelle C and colleagues conducted a study in French population of ALS patients. 289 patients were recruited for the study. All of them were randomly assigned the dose of Vitamin E and were assessed after every 3 months. The results did not show the effect in survival of muscle cells, except for the fact that patients who were administered the Vitamin E stayed in the milder form of disease for longer time [129]. Another study done in German population with high dose of Vitamin-E (5000 mg/day) showed ineffective results in comparison to the placebo effect [130]. However, in one of the meta-analyses of 23 studies published in year 2008, it was stated that antioxidants whether in combination or during individual administration do not show effective results [131]. Creatine one of the sports supplement has been known to increase the muscle strength. One study that came up in 1999 was conducted in animal model of ALS. Transgenic mice of ALS was administered with creatine dose. Authors reported that creatine was helpful in saving the mice neurons from dying in the age of 120 days. The group reported that creatine was also helpful in saving the mice from oxidative stress as well [132]. Later in 2004 a translational study performed with the same idea in human subjects demonstrated totally opposite results. 175 probable laboratory supported ALS patients were administered the 10 gm dose of creatine daily. The study showed no effect on survival rate neither it helped in reviving the rate of functional activities in patients [133]. Cannabinoid another naturally produced chemical present in humans as well as animals was studied by a group in 2004 in ALS mice model. They reported that Cannabinoid helped in prolonging survival of animals. Authors also reported reduced oxidative damage in spinal cord cell cultures of ALS mice and showed that it acts as an antiexcitotoxic agent *in vitro *[134]. Similar results have been shown in case of synthetically produced chemical called as cannabinol with dosage of 5 mg/kg/day for over a period of 12 weeks although no effect was there on survival [135]. Details for the functioning of these agents have not been mentioned but all of them report to choose or affect the oxidative stress pathway, although the oxidative stress based stimulation pathway by these compounds for *VEGF* cannot be ignored. Several studies report a common path for *VEGF* enhanced expression, but validity of usefulness of these natural components in case of ALS still needed to be studied. 

## 9. Concluding Remarks

The human, animal, and culture studies have shown that *VEGF* could be a promising therapeutic target in ALS. Upregulation of *VEGF* by different means such as genetic engineering, transplantation of stem cells overexpressing *VEGF*, and/or direct infusion of *VEGF* may rescue the damage of motor neurons and enhance the survival of patients with ALS either by increasing blood perfusion or direct neuroprotective effect on motor neurons. However, additional blinded preclinical studies of *VEGF*, particularly among primates, are still needed in ALS and other neurodegenerative disorders including Alzheimer's and Parkinson's disease before starting clinical trials. Regardless of the conflicting reports describing the role of oxidative stress and role of *VEGF* in various ALS investigations, both human and *in vivo* studies suffer from longitudinal analysis including the prospective nutritional interventional studies. Besides, the patient oriented genetic profiling studies have failed to include large cohort of homogeneous populations thus impacting the understanding of the demographic-SNP link in motor neuron degeneration. Nonpharmacological therapeutic approaches in ALS have not been adequately addressed and need new research focus for development of therapeutics.
